# Current research update on group B streptococcal infection related to obstetrics and gynecology

**DOI:** 10.3389/fphar.2024.1395673

**Published:** 2024-06-17

**Authors:** Ying Liu, Hao Ai

**Affiliations:** Liaoning Provincial Key Laboratory of Follicular Development and Reproductive Health, The Third Affiliated Hospital of Jinzhou Medical University, Jinzhou, Liaoning, China

**Keywords:** group B streptococcal, obstetrics and gynecology, antibiotic prophylaxis, group B streptococcal vaccine, microbial therapy

## Abstract

Group B streptococcal (GBS) is a Gram-positive bacterium that is commonly found in the gastrointestinal tract and urogenital tract. GBS infestation during pregnancy is a significant contributor to maternal and neonatal morbidity and mortality globally. This article aims to discuss the infectious diseases caused by GBS in the field of obstetrics and gynecology, as well as the challenges associated with the detection, treatment, and prevention of GBS.

## 1 Introduction of GBS

### 1.1 Microbiology

Group B streptococcal (GBS) is a Gram-positive, beta-hemolytic bacterium that appears as round or elliptical chains of cocci, usually in pairs or short chains, with a cell diameter of approximately 0.5–1.5 μm (Burcham et al., 2019). GBS is mainly classified based on its polysaccharide antigens, with at least ten different types of polysaccharide antigens identified (Tiruvayipati et al., 2021). The most common classification method is based on the capsular polysaccharide (CPS), which divides GBS into types Ia, Ib, II, III, IV, and others (Bianchi-Jassir et al., 2020). GBS is commonly found in the human digestive and reproductive tracts and typically ferments carbohydrates to produce lactic acid and carbon dioxide gas during the fermentation process (Goel et al., 2020). GBS requires a culture medium rich in blood components for growth and thrives in an acidic environment with a pH range of 5–6.5 (Bnfaga et al., 2023). GBS is sensitive to multiple antibiotics but may also exhibit some resistance (Koide et al., 2019). The genome size of GBS is approximately 2-3 Mbp. Its genome is a circular chromosome containing numerous coding and non-coding sequences. The structure and arrangement of the genome may vary among different strains. GBS exhibits genetic diversity, meaning that different strains may have distinct genome compositions and variations (Liu et al., 2023). The gene expression of GBS is influenced by complex regulatory networks, including transcription factors and other regulatory proteins, which help the bacterium adapt to and survive in different environments (Erickson Keesha et al., 2017). The GBS genome encodes many factors (Spencer et al., 2019) associated with pathogenicity, such as capsule polysaccharides, surface proteins, hemolysins, and enterotoxins. These factors play important roles in pathogenicity and the interaction with the host (Rajagopal, 2009).

Genomic analysis plays an important role in studying the genetic characteristics and pathogenic mechanisms of GBS (Schindler et al., 2023). Through sequencing technology (Preenanka and Safeena, 2023), the complete genome sequence of GBS can be obtained, which can then be used to study aspects such as genome structure, gene coding, and function. By comparing and analyzing the genome sequences of different strains, differences between different strains can be revealed, such as genome rearrangements and single nucleotide polymorphism (SNP) variations, and further research can be conducted on their relationship with pathogenicity. Transcriptome analysis techniques can be used to study changes in gene expression of GBS under different environmental conditions (Sitkiewicz et al., 2009), revealing its adaptability and biological characteristics. The pathogenic mechanisms of GBS include several aspects (Zadoks et al., 2011): the polysaccharide capsule of GBS is one of its main pathogenic factors. The capsule polysaccharide helps bacteria evade attacks from the host immune system and enhances their resistance to phagocytic cells, thereby increasing the chances of infection (Wang et al., 2022a). Surface proteins of GBS are also an important part of its pathogenic mechanisms (Xu et al., 2022). Surface proteins can bind to receptors on host cell surfaces, promoting bacterial adhesion and invasion. Some surface proteins also exhibit variability, making it more difficult for bacteria to be recognized and eliminated by the immune system. GBS produces hemolysins (Rosa-Fraile et al., 2014), which can destroy the membranes of host cells, leading to cell lysis and further promoting bacterial invasion and spread. GBS also causes inflammation through cell infiltration (Kuperwaser et al., 2023). It can stimulate host immune cells to release inflammatory mediators such as cytokines and chemokines, leading to tissue inflammation and damage. When infected with GBS, the host immune system produces specific antibodies and cellular immune responses. However, bacteria can interfere with host immune responses through various mechanisms, such as inhibiting cytokine production, evading phagocytosis by immune cells, and developing resistance, thereby enhancing their survival and reproduction. GBS is one of the main pathogens causing preterm birth and neonatal death (Le Gallou et al., 2023). GBS infections have certain epidemiological characteristics worldwide (Shabayek et al., 2018), influenced by factors such as geographic location, population demographics, and healthcare practices (Sidky and Thomas, 2002). The distribution and prevalence of GBS infections can differ significantly across various parts of the world, often due to environmental factors, climate, and the presence of specific GBS strains, which can affect local population susceptibility and the effectiveness of regional health strategies. Age distribution, genetic predispositions among certain populations, and socio-economic factors can influence the rate of GBS colonization and infection, leading to variations in disease incidence and outcomes among different demographic groups (Alizzi et al., 2022). The availability and implementation of screening and prevention measures, such as intrapartum antibiotic prophylaxis for GBS-positive pregnant women, greatly influence the incidence of neonatal GBS infections, with variations in healthcare quality and policies impacting overall disease management and outcomes (Schuchat, 1995). GBS is one of the main causes of preterm birth and neonatal death (Zhu and Lin, 2021). The main mode of transmission of GBS is vertical transmission (Mei et al., 2023), that is, transmission from an infected individual to a newborn or uninfected pregnant woman. Other modes of transmission include close contact transmission and healthcare-associated infections, but they are relatively rare. Under normal circumstances, the human immune system has a certain degree of protection against GBS (Korir et al., 2017). However, newborns and immunocompromised individuals are susceptible to infection. To prevent GBS infection, many countries and regions have implemented a series of preventive strategies, such as prenatal screening and prophylactic administration of antibiotics (Vieira et al., 2019).

The diagnostic methods of GBS are commonly used techniques in research and clinical practice. For the diagnosis of maternal infection, amniotic fluid samples can be cultured to detect the growth of GBS (Sayres et al., 2023). For screening of maternal infection, commonly used methods involve collecting vaginal and/or rectal samples for culture (Pierański et al., 2023). Screening before delivery is an important preventive strategy, especially for the diagnosis of neonatal infection, which can be detected through blood culture to determine the presence of GBS infection. Molecular biology techniques such as polymerase chain reaction (PCR) can detect the nucleic acid of GBS with high sensitivity and specificity (d'Otreppe et al., 2023). Understanding the susceptibility of GBS to antibiotics can guide the selection of clinical treatment (Husen et al., 2023). Commonly used antibiotic susceptibility testing methods include: disc diffusion method (Totadhri et al., 2022), where paper discs containing different antibiotics are placed on a culture medium to observe the relationship between bacterial growth and inhibition zones; broth dilution method (Stepanović et al., 2003), which gradually dilutes different concentrations of antibiotics in a culture medium to observe the minimum inhibitory concentration; E-test (Persson et al., 2008), which uses a gradient concentration of antibiotics on a strip to observe the relative position between bacterial growth and inhibition zones. These methods can be used to determine the susceptibility of GBS to a specific antibiotic, helping doctors choose appropriate drugs for treatment.

Preventing and controlling GBS infections is crucial for high-risk populations such as newborns and pregnant women. It is recommended to screen pregnant women for GBS colonization in the vagina and rectum, typically during late pregnancy (around 35–37 weeks). This can help detect the presence of GBS carriage and take appropriate preventive measures. For pregnant women who test positive for GBS carriage, it is advised to receive intravenous antibiotic prophylaxis during labor to reduce the risk of neonatal infection (Gurudas et al., 2022). Commonly used antibiotics include penicillin and ceftriaxone (Ali et al., 2022a), with specific antibiotic choices based on local treatment guidelines. If the mother is at risk of GBS infection, the newborn usually undergoes special observation and monitoring after birth. For high-risk newborns, antibiotic treatment may be needed to prevent infection. Necessary isolation and protective measures should be implemented in neonatal intensive care units or other settings prone to infection outbreaks to minimize the risk of pathogen transmission. Education and awareness campaigns about GBS infection should be conducted for healthcare workers, pregnant women, and families to enhance understanding and consciousness of prevention and control measures. Strengthening surveillance and reporting mechanisms, tracking the epidemiological characteristics of infection cases, and promptly implementing public health interventions are essential to reduce the spread and occurrence of GBS infections.

## 2 Virulence Factors

**FIGURE 1:**
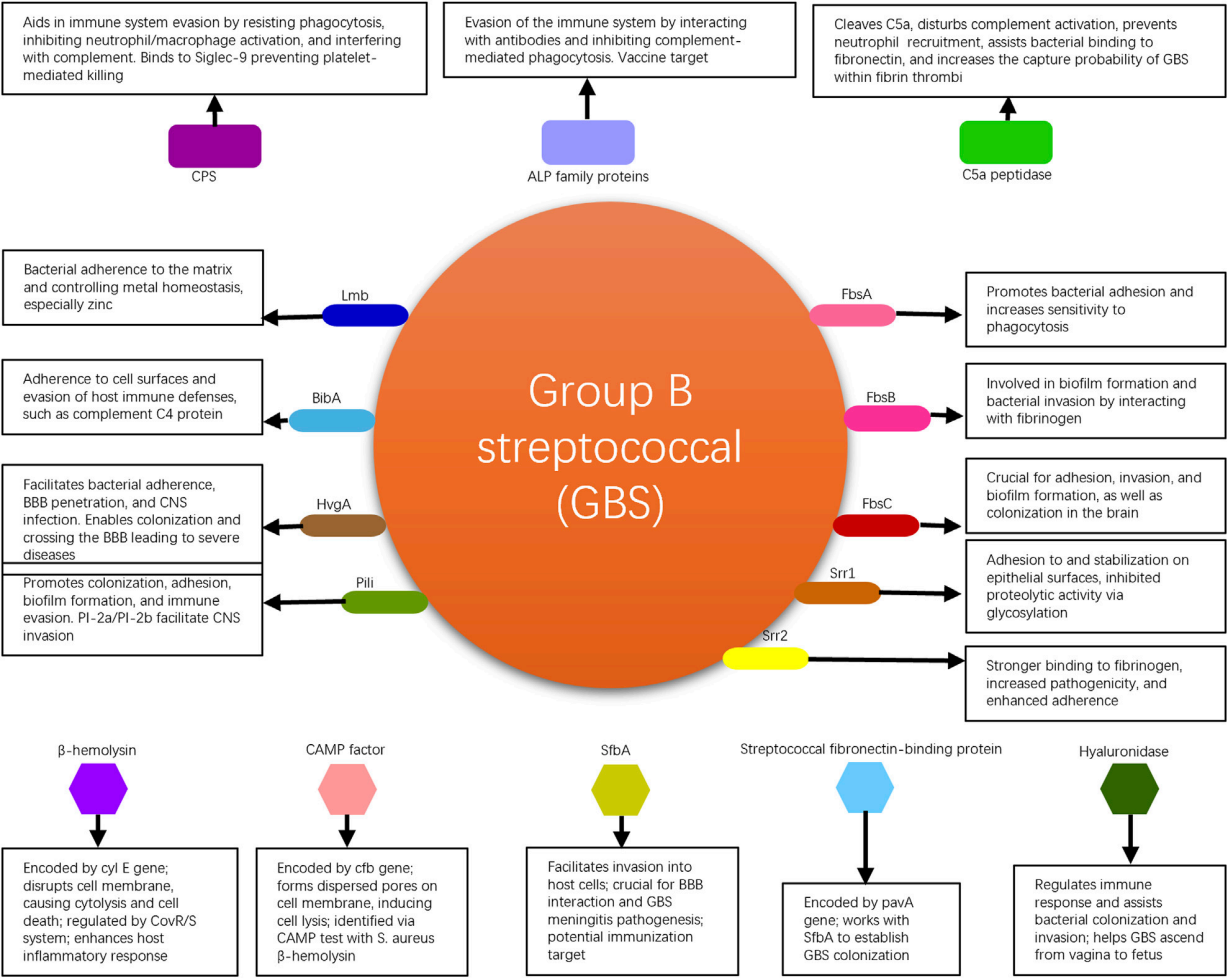
Summary of GBS virulence factors elucidated in this review, with their mechanisms.

GBS commonly colonizes the human genital tract and is one of the major pathogens during the perinatal period (Armistead et al., 2019). It can cause infections in pregnant women and, in severe cases, even jeopardize the lives of newborns. GBS possesses multiple virulence factors that are associated with bacterial adhesion, immune evasion, and invasive damage. These virulence factors enable the bacteria to persist within the human body, increasing the likelihood of transmission and worsening the infection, thereby affecting patient prognosis. GBS virulence factors elucidated in this review are shown in Figure 1.

### 2.1 Adherence-associated virulence factors

Fibrinogen-binding proteins (Fbs) are crucial proteins found on GBS (Buscetta et al., 2014). Three types of Fbs proteins have been identified: FbsA, FbsB, and FbsC. These proteins adhere to human skin cells to facilitate the colonization of GBS in the vaginal area. FbsA promotes bacterial adhesion to mucosal surfaces and increases their sensitivity to phagocytosis. FbsB is involved in the formation of bacterial biofilms and facilitates the invasion of lung epithelial cells by interacting with fibrinogen. Conversely, the loss of FbsC significantly impairs the adhesion, invasion, and biofilm formation abilities of the bacteria. FbsC, a pivotal factor in the brain colonization process by GBS, is notably absent in the notably aggressive ST17 strains, which are a sequence type known for their heightened virulence and strong association with serious neonatal infections, such as meningitis (Kardos et al., 2019; Yao et al., 2020). These findings are of significant importance in understanding the adhesion, invasion, and colonization mechanisms of GBS (Liu et al., 2022).

Serine-rich repeat proteins (Srr), which are rich in serine and characterized by amino acid sequence variations, can be divided into two subtypes, Srr1 and Srr2, in GBS (Chan et al., 2020). These proteins not only mediate invasion of endothelial cells by the bacteria but also assist in bacterial adherence by locking onto docking mechanisms. The process referred to as “locking onto docking mechanisms” implies the precise attachment or binding of these proteins to specific structures or receptors on the surface of host cells. This binding can be likened to inserting a key into a lock, where the Srr proteins (the “key”) have a specific molecular structure that allows them to securely attach to certain cell surface receptors or structures (the “dock”). This interaction facilitates bacterial adherence and invasion into host cells, thereby aiding the infection process. Through this precise docking mechanism, Srr proteins help to solidify the initial contact between GBS and the cells, further facilitating bacterial invasion and colonization. This mechanism is crucial not only for the pathogen’s adherence phase but also plays a role in its subsequent penetration through cellular barriers and dissemination within the host. Understanding this mechanism is therefore of significant importance for developing new strategies to combat pathogens that employ such mechanisms for infection. Most strains of GBS express Srr1, which promotes better adherence to the vaginal epithelium through its binding to human fibrinogen. Additionally, Srr1 enhances stability by inhibiting proteolytic activities through glycosylation, thereby prolonging bacterial adhesion and persistence. The stability enhanced by Srr1 refers to the structural and functional stability of the Srr1 protein itself on the surface of GBS bacteria. This stability is crucial for the prolonged adhesion and persistence of the bacteria on host tissues, such as the vaginal epithelium. Glycosylation of Srr1, a biochemical process in which a carbohydrate is covalently attached to the protein, plays a key role in this context. This glycosylation process can protect Srr1 from being degraded by proteolytic enzymes present in the host environment. Proteolytic enzymes are capable of breaking down proteins into peptides or amino acids, which could potentially disrupt the adherence mechanism of the bacteria to host cells. Therefore, by inhibiting proteolytic activities through glycosylation, Srr1 maintains its integrity and functionality longer, promoting a more stable bacterial adherence to host tissues. On the other hand, Srr2, a homologue of Srr1, is associated with the highly virulent clonal complex CC17. It exhibits stronger binding to human fibrinogen than Srr1 and strains expressing Srr2 are more pathogenic compared to those lacking Srr2. While Srr1 is expressed more abundantly in GBS, it cannot bind to plasminogen and plasmin, whereas Srr2 effectively interacts with them to enhance adherence strength. The interaction between bacterial surface proteins and host proteins plays a crucial role in the virulence of pathogens. In the case of GBS, the glycoproteins Srr1 and Srr2 have been identified as key players in adherence strength. While Srr1 is the most dominant glycoprotein, it is unable to bind to plasminogen and plasmin (Liu et al., 2022). On the other hand, Srr2 effectively interacts with plasminogen and plasmin, enhancing adherence strength (Liu et al., 2022). This difference in binding capabilities between Srr1 and Srr2 highlights the importance of specific protein interactions in bacterial pathogenicity. In a similar context, *Staphylococcus aureus* has been shown to utilize adhesive virulence factors to resist host defenses. The staphylokinase (SAK) protein interacts with the serine protease domain of plasmin, enhancing resistance to digestion (Risser et al., 2022). This interaction with plasmin is crucial for the pathogen’s ability to evade host immune responses. Additionally, the molecular interactions of human plasminogen with fibronectin-binding proteins further emphasize the significance of protein-protein interactions in bacterial adherence and virulence (Risser et al., 2022). Overall, the ability of bacterial surface proteins to interact with host proteins such as plasminogen and plasmin is a key determinant of pathogenicity. While some proteins like Srr1 lack the ability to bind to these host proteins, others like Srr2 can effectively interact with them to enhance adherence strength. The structures of Srr1 and Srr2 are highly conserved in GBS, and vaccination with the corresponding “latch peptide” has been shown to provide serotype-independent protection against relevant infections in mice (Lin et al., 2017).

The laminin-binding protein (Lmb) (Spellerberg et al., 1999) in GBS, encoded by the *lmb* gene, facilitates adherence of the bacteria to extracellular matrix molecules in the human body and binds to the major component of the basement membrane, laminin. Lmb participates in the regulation of intracellular metal homeostasis by coordinating zinc ions with histidine residues to form a tetrahedral structure. This enables the control of zinc influx and efflux in bacterial cells, thereby prolonging survival in the human body and promoting pathogenicity. Bacteria lacking Lmb not only exhibit reduced invasiveness towards human brain microvascular endothelial cells and impaired neurotropism but also display decreased resistance to zinc ions. Lmb mediates the attachment of GBS to human laminin, facilitating bacterial colonization and invasion (Liu et al., 2022). The *lmb* gene encodes Lmb, which plays a crucial role in binding to laminin, a component of host cells, thereby increasing GBS’s pathogenic potential (Armistead et al., 2019). Additionally, Lmb promotes GBS adherence to host tissues, reflecting changes in GBS pathogenicity (Upadhyay et al., 2022). Studies have shown that Lmb, along with other virulence factors such as hypervirulent GBS adhesin (HvgA), contributes to the high pathogenicity of certain GBS strains (Shimizu et al., 2020; Kamińska et al., 2024). Furthermore, Lmb is identified as an immunogenic protein of GBS, interacting with host immune cells and potentially modulating host immune responses (Dobrut and Brzychczy-Włoch, 2022). The crystal structure of Lmb has been elucidated, providing insights into its function and potential as a target for therapeutic interventions (Ragunathan et al., 2013). Overall, the laminin-binding protein Lmb is a critical virulence factor in GBS pathogenicity, highlighting its importance in the colonization and invasion processes of this pathogen (Liu et al., 2019; Lacasse et al., 2022).

The immunogenic bacterial adhesin (BibA) (Santi et al., 2007) is a cell wall-anchored protein produced by GBS that promotes bacterial adherence to the surface of human cervical and lung epithelial cells. This protein can also interfere with the host’s antimicrobial defense mechanisms, such as phagocytosis by white blood cells, by regulating the interaction between the bacteria and complement C4-binding protein, thereby aiding the survival of GBS in the bloodstream. A report suggest that BibA is a strong and specific vaccine target. It demonstrated in a mouse model that a vaccine formulation containing BibA induced the production of protective antibodies against GBS, which could help prevent vaginal colonization and invasive infections caused by this bacterium (Dos Santos et al., 2020).

The hypervirulent GBS adhesin (HvgA) is a cell wall-anchored protein specific to the highly pathogenic clone CC17 of GBS (Li et al., 2019). It is closely associated with the development of late-onset diseases (LOD), such as neonatal meningitis (Pietrocola et al., 2018). Enhanced expression of HvgA facilitates bacterial adherence to intestinal epithelial cells, choroid plexus epithelial cells, and microvascular endothelial cells that constitute the blood-brain barrier (BBB). In a mouse experiment, HvgA-expressing GBS showed greater ability to colonize and penetrate the blood-brain barrier compared to strains lacking HvgA, leading to severe consequences. This suggests that GBS, under the mediation of HvgA, can breach the blood-brain barrier and cause central nervous system infections (Kekic et al., 2021).

The pili (PI) of GBS are considered essential structures for promoting bacterial colonization, biofilm formation, and central nervous system invasion (Danne and Dramsi, 2012). The genes encoding these pili are categorized into two types: *Pili-1* (*PI-1*) and *Pili-2* (*PI-2*). Among them, *PI-2* is further divided into two subtypes, *PI-2a* and *PI-2b*. While the genes for pili may be present in varying degrees in the bacterial genome, a single strain of GBS may express only one type of pili. The GBS pili consist of three structural protein subunits: pili associated adhesin (PilA) at the tip, pili shaft backbone protein (PilB), and pili anchor (PilC) at the base. Research has shown that PilA enhances bacterial adherence to vaginal and cervical epithelial cells (Pezzicoli et al., 2008), while the biofilm synthesized by PilB is involved in bacterial invasion and resistance to phagocytosis (Maeda et al., 2021). One study has found that since almost all GBS strains possess pili, a vaccine containing conserved components of the pili island would provide high-level protection against the majority of GBS strains (Margarit et al., 2009).

### 2.2 Bacterial immune evasion related virulence factors

The capsular polysaccharide (CPS) of GBS aids in bacterial colonization and survival in the human body. CPS is an important virulence factor that mediates immune evasion. Its specificity is determined by the specific arrangement of sugars within each polysaccharide repeat unit. GBS can be classified into 10 CPS serotypes (Ia, Ib, II-IX). All 10 serotypes can cause disease, although the types and rates of disease vary among different serotypes (Lin E. et al., 2021). The distribution of CPS serotypes is influenced by factors such as geographic region and ethnicity (Wu et al., 2019). CPS not only resists phagocytosis by immune cells but also inhibits the activation of neutrophils and macrophages, thereby helping the bacteria evade the immune defenses of the host. It also promotes biofilm formation and interferes with complement defense, playing an important role in the infection process. GBS CPS contains α2,3-linked sialic acid residues (Sia), which effectively inhibit platelet-mediated killing of GBS, counteract antibacterial components produced by platelets, and can bind to the Siglec-9 receptor on the surface of platelets, thus inhibiting platelet activation (Armistead et al., 2019). CPS is an important target for vaccine development. Monovalent vaccines designed based on common CPS serotypes (Ia, Ib, II, III, and V) have entered phase I clinical trials (Baker et al., 1999; Baker et al., 2000; Baker et al., 2003; Baker et al., 2004). Trivalent vaccines targeting serotypes Ia, Ib, and III have shown high levels of specificity, safety, and tolerance in infants (Madhi et al., 2017; Swamy et al., 2020). A hexavalent vaccine containing serotypes Ia, Ib, II, III, IV, and V has been developed by BUURMAN et al. and it is the most comprehensive vaccine to date, including the largest number of serotypes (Buurman et al., 2019). Animal experimental results have shown that this hexavalent vaccine has a good immunogenicity and is expected to apply for clinical trials.

ALP family proteins are commonly expressed virulence factors in GBS that are also associated with immune evasion (Paoletti and Kasper, 2019; Lin L. et al., 2021). This family of proteins includes ALP-C, ALP-1, ALP-2, ALP-3, ALP-4, and Rib, encoded by the genes *bca*, *alp1*, *alp2*, *alp3*, *alp4*, and *rib*, respectively, and their amino acid sequences exhibit homology (Furfaro et al., 2018). A study has found that antibodies designed against ALP family proteins in mouse models attenuate infections caused by homologous GBS strains, indicating that loss of the repetitive gene sequences in this protein family is a mechanism by which bacteria interact with and evade the human immune system (Paoletti and Kasper, 2019). Beta-C protein, which is similar to ALP-C and encoded by the *bac* gene, can bind to IgA antibodies and inhibit complement-mediated phagocytosis. Immunizing pregnant mice with this protein immunogen protects newborn mice from invasive GBS infection, possibly by accelerating the phagocytosis of bacteria by white blood cells (Zastempowska et al., 2022). On the other hand, since over 90% of GBS strains express one or more proteins from this family, the ALP protein family is a highly specific vaccine target (Gabrielsen et al., 2017). Vaccines based on the highly immunogenic N-terminal domain of ALP-C and Rib (GBS-NN) have completed phase I clinical trials, resulting in over 30 times increase in GBS-specific antibodies in the sera of 240 female participants (Lin et al., 2018).

Streptococcal C5a peptidase from GBS is encoded by the *scpB* gene and is a serine protease. It can cleave the neutrophil chemoattractant C5a, thereby interrupting complement activation. It also functions as an allergenic toxin involved in the invasion of epithelial cells (Shabayek et al., 2018), inhibits neutrophil recruitment (Tulyaprawat et al., 2021), and aids in GBS binding to fibronectin, facilitating the invasion of human epithelial cells (Liu et al., 2022). Bone marrow-derived mast cells (BMMC) contain abundant factor XIIIA (FXIIIA), which has been recently demonstrated to crosslink fibrinogen through the contribution of *scpB* gene, increasing the capture probability of GBS within fibrin thrombi and assisting in host defense against GBS infection (Piliponsky et al., 2022). C5a peptidase is highly conserved and widely expressed in GBS. Researchers further evaluated the potential as a vaccine antigen by using a mouse model. They encapsulated C5a peptidase in microspheres and inoculated mice, finding that mice immunized with C5a peptidase encapsulated microspheres exhibited a high immune response against GBS, and the mortality rate was significantly reduced compared to mice not receiving C5a peptidase encapsulated microspheres (Santillan et al., 2008; Santillan et al., 2011).

### 2.3 Bacterial invasion associated virulence factors

GBS belonging to beta-hemolytic streptococci produces β-hemolysin encoded by the *cyl E* gene and the CAMP factor encoded by the *cfb* gene, which cause various tissue damage by lysing human cells (Liu et al., 2022). β-hemolysin itself possesses lytic properties, disrupting cell membrane structure and function, leading to cytolysis and cell death. Transcription of the *cyl E* gene and production of hemolysin are negatively regulated by the CovR/S two-component system, promoting the release of inflammatory factors by host cells to enhance bacterial damage to the host (Koo et al., 2019). The CAMP factor aggregates on the cell membrane surface, forming dispersed pores that induce cell lysis. A crucial phenotypic test used in clinical laboratories to identify GBS is the CAMP test, which is based on the synergy between the CAMP factor and β-hemolysin from *Staphylococcus aureus*, resulting in the lysis of blood cells and the formation of a characteristic arrowhead-shaped hemolytic zone (Liu et al., 2022).

Streptococcal fibronectin-binding protein A (SfbA) is highly conserved in GBS and facilitates the invasion of GBS into human vaginal and cervical cells, brain microvascular endothelial cells, and astrocytes, but it does not enhance GBS adhesion to host cells (Shabayek et al., 2018). Additionally, SfbA plays a crucial role in the interaction between GBS and the blood-brain barrier and in the pathogenesis of GBS meningitis. Immunization targeting SfbA can help prevent neonatal GBS meningitis infection (Mu et al., 2014). The fibronectin-binding protein encoded by the *pavA* gene is an extracellular surface protein of GBS that is involved in GBS colonization. This protein, along with SfbA, contributes to GBS colonization and establishment of the ecological niche in the vagina (Gendrin et al., 2018; Yoshida et al., 2021). The pathogenesis of streptococcal infections is a complex process involving various virulence factors and regulatory mechanisms. One such factor is the fibronectin-binding protein A gene, which plays a crucial role in the adherence of streptococci to host cells. A study has shown that disruption of genes encoding fibronectin-binding proteins can reduce bacterial adherence to human endothelial cells (Deng et al., 2019). Additionally, fibronectin-binding proteins have been implicated in promoting inflammation during the pathogenesis of meningitis caused by *streptococci* (Deng et al., 2019).

Hyaluronidase, an extracellular enzyme released by GBS, is encoded by the *hylB* gene. This enzyme degrades hyaluronic acid polymers, which are present in the extracellular matrix of human cells, into disaccharide units, disrupting cellular signaling and promoting the expression of inflammatory mediators. It has the capability to break down hyaluronic acid in the connective tissue matrix, disintegrate proteoglycans in connective tissues, and regulate the immune response during colonization and invasion by the bacteria, suppressing the production of reactive oxygen species (ROS) and resisting the action of neutrophils (Coleman et al., 2021; Kurian and Modi, 2022). Most importantly, hyaluronidase can breach the barrier between mother and fetus, allowing GBS to ascend from the vagina to the fetus, leading to fatal infections in the fetus (Liu et al., 2022). GBS virulence factors with their specific targets and mechanisms are shown in Table 1.

**TABLE 1:** Summary of GBS virulence factors with their specific targets, mechanisms and references.

Virulence factor	Specific target	Mechanism	References
FbsA	Mucosal surfaces	Promotes bacterial adhesion and increases sensitivity to phagocytosis	Buscetta et al. (2014)
FbsB	Fibrinogen, lung epithelial cells	Involved in biofilm formation and bacterial invasion by interacting with fibrinogen	Buscetta et al. (2014)
FbsC	Various bodily sites, brain	Crucial for adhesion, invasion, and biofilm formation, as well as colonization in the brain	Kardos et al. (2019), Yao et al. (2020)
Srr1	Human fibrinogen, vaginal epithelium	Adhesion to and stabilization on epithelial surfaces, inhibited proteolytic activity via glycosylation	Chan et al. (2020), Liu et al. (2022)
Srr2	Human fibrinogen, plasminogen, plasmin	Stronger binding to fibrinogen, increased pathogenicity, and enhanced adherence	Chan et al. (2020), Liu et al. (2022)
Lmb	Laminin, extracellular matrix	Bacterial adherence to the matrix and controlling metal homeostasis, especially zinc	Spellerberg et al. (1999)
BibA	Cervical and lung epithelial cells	Adherence to cell surfaces and evasion of host immune defenses, such as complement C4 protein	Santi et al. (2007), Dos Santos et al. (2020)
HvgA	Intestinal epithelial cells, choroid plexus epithelial cells, microvascular endothelial cells	Facilitates bacterial adherence, BBB penetration, and CNS infection. Enables colonization and crossing the BBB leading to severe diseases	Pietrocola et al. (2018), Li et al. (2019), Kekic et al. (2021)
Pili (PI-1/PI-2)	Vaginal and cervical epithelial cells, central nervous system	Promotes colonization, adhesion, biofilm formation, and immune evasion. PI-2a/PI-2b facilitate CNS invasion	Pezzicoli et al. (2008), Margarit et al. (2009), Danne and Dramsi (2012), Maeda et al. (2021)
CPS	Immune system cells, biofilm formation, complement defense, platelets	Aids in immune system evasion by resisting phagocytosis, inhibiting neutrophil/macrophage activation, and interfering with complement. Binds to Siglec-9 preventing platelet-mediated killing	Baker et al. (1999), Baker et al. (2000), Baker et al. (2003), Baker et al. (2004), Madhi et al. (2017), Buurman et al. (2019), Wu et al. (2019), Swamy et al. (2020), Lin et al. (2021a)
ALP family proteins	Immune system cells	Evasion of the immune system by interacting with antibodies and inhibiting complement-mediated phagocytosis. Vaccine target.	Gabrielsen et al. (2017), Furfaro et al. (2018), Lin et al. (2018), Paoletti and Kasper (2019), Lin et al. (2021b), Zastempowska et al. (2022)
C5a peptidase	Neutrophils, human epithelial cells, BMMC (factor FXIIIA)	Cleaves C5a, disturbs complement activation, prevents neutrophil recruitment, assists bacterial binding to fibronectin, and increases the capture probability of GBS within fibrin thrombi	(Shabayek et al., 2018; Liu et al., 2022; Santillan et al., 2008; Santillan et al., 2011; Tulyaprawat et al., 2021; Piliponsky et al., 2022)
β-hemolysin	Human cells (tissue damage)	Encoded by cyl E gene; disrupts cell membrane, causing cytolysis and cell death; regulated by CovR/S system; enhances host inflammatory response	(Liu et al., 2022; Koo et al., 2019)
CAMP factor	Human cells (tissue damage)	Encoded by cfb gene; forms dispersed pores on cell membrane, inducing cell lysis; identified via CAMP test with *S. aureus* β-hemolysin	Liu et al. (2022)
SfbA	Vaginal and cervical cells, brain microvascular endothelial cells, astrocytes; Vaginal niche colonization	Facilitates invasion into host cells; crucial for BBB interaction and GBS meningitis pathogenesis; potential immunization target; Encoded by pavA gene; works with SfbA to establish GBS colonization	(Shabayek et al., 2018; Mu et al., 2014; Gendrin et al., 2018; Deng et al., 2019; Yoshida et al., 2021)
Hyaluronidase	Extracellular matrix, connective tissue, Immune response, mother-fetus barrier	Regulates immune response and assists bacterial colonization and invasion; helps GBS ascend from vagina to fetus	(Liu et al., 2022; Coleman et al., 2021; Kurian and Modi, 2022)

## 3 Antibiotic resistance in GBS

GBS poses considerable risks for both expectant mothers and their babies. In pregnant women, it can lead to serious infections such as sepsis, inflammation of the fetal membranes known as chorioamnionitis, and postpartum endometritis. Additionally, GBS can cause adverse pregnancy outcomes, including premature rupture of membranes, miscarriage, preterm delivery, and intrauterine growth restriction. Late-pregnancy colonization by GBS stands as a major threat for neonatal infection (Chattopadhyay et al., 2011), with approximately 1%–2% of newborns from GBS colonization-positive mothers contracting invasive infections (Yu et al., 2011). These newborns primarily suffer from sepsis and meningitis that are both aggressive and life-threatening. Consequently, such infections have high mortality and disability rates, jeopardizing the health and wellbeing of the affected neonates.

The implementation of intrapartum antibiotic prophylaxis (IAP) strategies has significantly reduced the incidence and adverse impacts of perinatal GBS infections in European and American countries (MMWR, 1997). However, the overuse of antibiotics in recent years has led to growing concerns about the emergence of antibiotic-resistant GBS strains on a global scale. Understanding the resistance patterns of GBS is of critical importance for guiding the rational use of antibiotics in clinical settings.

### 3.1 Penicillin resistance in GBS and its underlying mechanisms

Compared to the high resistance rates observed with erythromycin and clindamycin, numerous studies have confirmed that GBS retains high sensitivity towards penicillin, which remains the preferred drug for the prophylactic treatment of GBS infections (Verani et al., 2010). However, with the rising use of antibiotics, a change in sensitivity has been detected. Since 1994, reports of GBS strains with reduced penicillin susceptibility (PRGBS) have emerged sporadically. Since 2008, there has been evidence suggesting an increasing trend in the minimum inhibitory concentration (MIC) values of penicillin against GBS, indicating a tendency towards resistance (Kimura et al., 2008; Nagano et al., 2012). PRGBS resistant to beta-lactam antibiotics is an important emerging problem. Cases of PRGBS have been reported in regions such as Hong Kong (Chu et al., 2007), the United States (Dahesh et al., 2008), Canada (Longtin et al., 2011), and Japan (Kimura et al., 2008), with MIC values reaching from 0.25 to 1.00 mg/L. In Japan, studies have shown a high isolation rate of multidrug-resistant (MDR) group B streptococci with reduced penicillin susceptibility in Japan, indicating a growing issue with antibiotic resistance in this region (Ali et al., 2022b; Koide et al., 2022; Khan et al., 2023; Verma et al., 2023). Currently, the mechanisms underlying the reduced sensitivity of GBS to penicillin are not fully understood. Japanese researchers have attributed the decrease in penicillin susceptibility to mutations in the genes encoding penicillin-binding proteins (PBPs), specifically *PBP1A*, *PBP2B*, and *PBP2X* genes [101–103]. Notably, amino acid substitutions V405A and Q557E in *PBP2X* gene have been found to form unstable proteins, leading to a reduction and weakened affinity of the associated penicillin-binding proteins, which is a major mechanism of decreased penicillin sensitivity in GBS (Uruén et al., 2022). Moreover, multiple amino acid substitutions in PBPs 2X, 2B, and 1A have been discovered (Kimura et al., 2008; Nagano et al., 2012). Research in Canada on PRGBS identified amino acid substitutions in multiple PBPs but did not find the V405A and Q557E substitutions in *PBP2X* gene (V405A refers to a substitution where valine (V) at position 405 in the protein sequence is replaced by alanine (A), and Q557E refers to a substitution where glutamine (Q) at position 557 is replaced by glutamic acid (E)) (Longtin et al., 2011).

### 3.2 Resistance to erythromycin and clindamycin in GBS and its underlying mechanisms

Penicillin is the front-line treatment for both prevention and management of GBS infections. For those allergic to penicillin, clindamycin and erythromycin serve as the primary alternatives and are used by approximately 20% of GBS carriers. With the increasing use of these drugs, there has been a global rise in resistance to erythromycin and clindamycin (Savoia et al., 2008; Sadowy et al., 2010).

The resistance rate of GBS among pregnant women has been a growing concern in recent years. Studies from various regions have reported high rates of antimicrobial resistance in GBS isolates. Du et al. (Du et al., 2021) found that in Vietnamese pregnant women, the multidrug-resistance rate was 59.19%, with 8.46% of isolates resistant to six to seven antibiotics. Similarly, Bae et al. (Bae et al., 2022) reported a nationwide GBS colonization rate of 10.6% in pregnant Korean women. Furthermore, Du et al. (Van Du et al., 2021) highlighted the importance of considering the high rates of erythromycin, clindamycin, and multidrug resistance in GBS as a risk factor for neonates. This is supported by Hsu et al. (Hsu et al., 2023), who found that serotype Ib GBS strains had significantly higher rates of resistance to erythromycin and clindamycin compared to other serotypes. Moreover, Wang et al. (Wang et al., 2023) conducted a systematic review and meta-analysis in China, indicating a concerning emergence of penicillin resistance among GBS strains. This aligns with the findings of Verma et al. (Verma et al., 2023), who reported the highest resistance rate for penicillin among all tested antibiotics in GBS isolates of Indian origin.

GBS exhibits resistance to macrolide(M), clindamycin(L), and Streptogramin B(SB), together classified as the MLS group, encompassing three distinct yet functionally related types of antibiotics. There are three predominant mechanisms of GBS resistance to macrolide antibiotics:

M Phenotype Resistance: The resistance mechanism involves active efflux, where efflux pumps extrude the antibiotic out of the cell, leading to resistance. The efflux pump-related proteins are encoded by *mef* genes, which confer resistance to 14- and 15-membered ring macrolides but sensitivity to 16-membered macrolides, clindamycin, and Streptogramin B. This typically results in moderate-level resistance, with erythromycin MIC ranging from 1 to 32 mg/L. The *mefA* gene, one of two subtypes of the *mef* gene, is located on the Tn1207.1 transposon in pyogenic streptococci (Bacciaglia et al., 2007).

MLSB Phenotype Resistance: The mechanism involves an alteration in ribosomal target sites, primarily mediated by *erm* genes encoding ribosomal methylases that methylate a single adenine residue in 23SrRNA. This methylation reduces the affinity of the macrolide antibiotics to the ribosomal binding sites (Lopardo et al., 2005). *erm* gene-mediated macrolide resistance is generally of a high level, with erythromycin MIC values exceeding 256 mg/L, and cross-resistance occurs with clindamycin and Streptogramin B. The MLSB phenotype is divided into constitutive (cMLSB) and inducible (iMLSB) types. cMLSB occurs when *erm* genes are stably expressed, which results in resistance to erythromycin, clindamycin, and other MLS group members. iMLSB relates to scenarios where the *erm* genes require inducers to express resistance to clindamycin; erythromycin can act as such an inducer. Otherwise, clindamycin sensitivity might appear *in vitro* tests (Shen et al., 2005).

Both the M phenotype resistance and iMLSB resistance appear with erythromycin resistance but clindamycin sensitivity (Akdoğan Kittana et al., 2019). The presence of erythromycin ribosome methylase (*erm*) genes has been linked to the expression of inducible clindamycin resistance in *Staphylococcus aureus* (Heyar et al., 2020). However, the prevalence of iMLSB phenotype may vary depending on the study population, with lower rates observed in rural areas where antimicrobial exposure is limited (Heyar et al., 2020). In clinical settings, it is crucial to accurately identify clindamycin resistance, as studies have shown that a significant proportion of *Staphylococcus aureus* isolates can exhibit inducible clindamycin resistance, which may be misidentified as clindamycin susceptible using standard methods (Padekar et al., 2020). The cMLSB phenotype has been reported as the predominant form of resistance, followed by the iMLSB phenotype in some studies (Kumar Chaudhary and Piya, 2021). To differentiate these two phenotypes, the National Committee for Clinical Laboratory Standards (NCCLS) in the United States recommended the D-test in 2004. This involves placing a clindamycin disk (2 μg/disk) 20 mm away from an erythromycin disk (15 μg/disk), incubating at 35°C for 16–18 h. A “D” shape flattening or blunting of the inhibition zone adjacent to the erythromycin disc indicates a positive D-test, suggesting inducible clindamycin resistance (iMLSB type); otherwise, the test is negative (M type resistance) (Back et al., 2012). The D-test distinguishes iMLSB resistance and corrects clindamycin sensitivity results, aiding in rational pharmacotherapy.

L Phenotype Resistance: This resistance is due to adenylation. Enzymes encoded by the *linB* and *lnu* genes mediate the inactivation of lincosamide antibiotics (de Azavedo et al., 2001; Faccone et al., 2010; Seo et al., 2010). It is characterized by sensitivity to erythromycin and resistance to clindamycin. Studies such as by Lu et al. indicated that 4.5% of GBS strains are L phenotype resistant (Lu et al., 2014), with the prevalence of the *linB* gene being significantly lower than reported in Korea, suggesting geographical variation in L phenotype resistance and *linB* gene carriage. The *linB* gene, which is linked to clindamycin resistance, can lead to an L phenotype, conferring resistance to lincosamides only (Santana et al., 2020). The presence of antibiotic resistance genes, such as *linB*, in GBS strains highlights the importance of monitoring and understanding geographical variation in resistance patterns to inform treatment strategies and vaccine design (Santana et al., 2020; Barros, 2021; Mudzana et al., 2021). The inactivation of lincosamide antibiotics mediated by the *lnu* gene was first reported in *Enterococcus faecium HM1025* (Bozdogan et al., 1999). L phenotype resistance regulated by the *lnuB* gene has been documented in various regions, including Latin America, Canada, Korea, and Spain (Arana et al., 2014).

### 3.3 Mechanisms of GBS resistance to telithromycin

A study from the United States between 2001 and 2004 indicated a 53.5% resistance rate to erythromycin in GBS, while the non-susceptibility rate for tetracycline was only 1.5% (DiPersio and DiPersio, 2006). Research in China has shown that among pregnant women colonized with GBS, non-susceptibility rates for erythromycin, clarithromycin, and azithromycin all exceeded 85.0%, while for tetracycline it was only 31.0%. This suggests a sensitivity to tetracycline despite resistance to other macrolides (Wang et al., 2015).

Telithromycin, the first ketolide and a 14-membered ring macrolide, demonstrates a strong affinity towards bacterial ribosomes, enabling it to counteract common macrolide antibiotic resistance mechanisms. These mechanisms include methyltransferase enzymatic activity encoded by the *ermB* gene, which results in the dimethylation of an adenine residue at the N-6 position on the 23SrRNA, and ribosomal protein variations that interfere with the binding of macrolides to bacteria. Telithromycin has been proven to exhibit greater antimicrobial activity against erythromycin-resistant strains, as confirmed in *Streptococcus pneumoniae* (Farrell and Felmingham, 2004; Takaya et al., 2010).

### 3.4 Resistance to fluoroquinolone antibiotics in GBS and the mechanisms involved

In 2003, Japan first reported the isolation of fluoroquinolone-resistant GBS strains, although the initial rate was low. Then several countries and regions have reported the emergence of GBS isolates resistant to fluoroquinolones (Ali et al., 2020; Hayes et al., 2020). In Taiwan the resistance rate to quinolones ranges between 0.3% and 5.0%, and all GBS strains resistant to levofloxacin also exhibited higher MIC values for ciprofloxacin, gatifloxacin, moxifloxacin, and gemifloxacin (Back et al., 2012). In 2014, Italy first reported the presence of levofloxacin-resistant GBS strains with a resistance rate of 1.4% (Piccinelli et al., 2015a), and another study in the same year reported a resistance rate of 3.4% (Piccinelli et al., 2015b). Mutations in the quinolone resistance-determining regions (QRDRs) of genes encoding the topoisomerase IV subunit C (ParC) and the DNA gyrase subunit A (GyrA) have been closely associated with GBS resistance to fluoroquinolone antibiotics (Wehbeh et al., 2005; Murayama et al., 2009). Double mutations in GyrA Ser-81 to Leu and in ParC Ser-79 to Phe or Tyr are associated with high-level resistance to levofloxacin. Additional mutations have been discovered in ParC, such as Asp-83 to Tyr and Asp-83 to Asn. Similar mutations have also been found in GyrB, but their significance is yet to be clarified (Piccinelli et al., 2015b). Clinical isolates of GBS resistant to levofloxacin have been reported to belong predominantly to clonal complex III/ST19. Wang et al. found that the resistance rate of III/ST19 GBS strains to levofloxacin reached 92.9%, with 75% of the levofloxacin-resistant strains belonging to CC19, whereas all III/ST17 type GBS strains were sensitive to levofloxacin (Wang et al., 2013). Research in Italy indicated that the majority of levofloxacin-resistant GBS strains were of the Ib/ST19 type, also within the CC19 (Piccinelli et al., 2015b). It is speculated that the sensitivity of levofloxacin in bacteria may be related to their molecular biological characteristics and serotype, suggesting possible clonal spread.

### 3.5 Resistance to tetracycline in GBS and the underlying mechanisms

Research both nationally and internationally has consistently shown high rates of resistance to tetracycline in GBS. Resistance rates reported include 62% in Canada, 80% in Italy, 97% in Brazil, and 98% in Egypt (Shabayek and Abdalla, 2014; Piccinelli et al., 2015b). The tetracycline resistance gene primarily involves *tetM* (Granlund et al., 2010), which encodes ribosomal protection proteins. In China, *tetM* and *tetO* genes are the main tetracycline resistance genes found in GBS, with the detection of *tetK* and *tetL* genes also reported (Huiling et al., 2010; Jia-de, 2010). In Brazil, resistance is predominantly due to the *tetM* gene (99.3%), with a 1.8% carriage rate for *tetO* (Dutra et al., 2014). In Egypt, *tetM* is also the main resistance gene, with an individual carriage rate of 83.7%, and the presence of *tetL*, *tetK*, and *tetO* genes has been detected (Shabayek and Abdalla, 2014). Tetracyclines are known to affect the development of teeth and bones in children, and due to concerns about severe hepatorenal toxicity reactions, its use has been largely discontinued in pediatric clinical practice for many years. However, the problem of tetracycline resistance remains very serious in China (Liu et al., 2021). This issue may be related to the overuse of these antibiotics in agriculture and food animals, as well as the stable resistance of bacteria to this class of antibiotics. Further investigation is warranted into this matter.

### 3.6 Vancomycin resistance in GBS

Due to rising resistance rates to erythromycin and clindamycin, vancomycin is sometimes necessary for the prevention and treatment of GBS infections in patients allergic to penicillin. Park et al. explored two laboratory-confirmed cases of invasive GBS strains resistant to vancomycin (Park et al., 2014). This study employed PCR amplification with primers, EG1 and, EG2 to produce a sequence similar to the vanG (941bp) of *Enterococci* and confirmed that the strains contained sequences corresponding to vanW, vanG and vanXY, with sequence similarities of 89.8%, 91.0%, and 95.7%, respectively. One of the isolates had a 2658bp tandem repeat sequence completely identical to the vanG of *Enterococcus faecalis*. Since there was no epidemiological link between the strains, it is conjectured that independent mechanisms of resistance acquisition exist. Further research is needed, in conjunction with clinical outcomes, to investigate their origins and patterns of spread.

### 3.7 Multidrug resistance in GBS

In recent years, the problem of drug resistance in GBS has become increasingly serious globally, with reports emerging of multidrug-resistant GBS strains (Talebi Bezmin Abadi et al., 2019). Additionally, a study revealed an increasing trend in macrolide-resistant GBS isolates (Khan et al., 2023). PRGBS is capable of surviving and spreading in hospital settings, leading to nosocomial infections. There is a potential risk of global transmission and epidemic spread in the future.

GBS colonization is a significant risk factor for various adverse outcomes in pregnant women and neonates. Studies have shown that GBS colonization in the vaginal tract is associated with preterm birth (Tano et al., 2021) and neonatal GBS early-onset disease (Zhu and Lin, 2021). The prevalence of GBS colonization varies depending on the detection method used, with enrichment media improving the detection rate (Song et al., 2022). In the context of GBS colonization and infection, alternative antimicrobials such as cefazolin have been explored as prophylactic regimens, especially in situations where penicillins are contraindicated or unavailable (Antonello et al., 2020). Additionally, the relationship between the gut microbiota composition in pregnant women colonized with GBS and maternal blood routine as well as neonatal blood-gas analysis has been investigated to understand the interplay between GBS colonization and adverse birth outcomes (Wang et al., 2022b). Furthermore, the prevalence and clinical relevance of colonization with methicillin-resistant *Staphylococcus aureus* (MRSA) in the obstetric population have been studied to assess the potential impact on both mother and child (Bauters et al., 2022). Maternal GBS colonization has been identified as a major risk factor for neonatal GBS infection, emphasizing the importance of understanding and addressing GBS colonization in pregnant women (Jung et al., 2021).

The epidemiology of multidrug-resistant GBS remains a significant concern globally, with studies focusing on different aspects of this pathogen. Huang et al. (Huang et al., 2019) reviewed data from China to determine the maternal GBS colonization rate, incidence of invasive GBS disease in infants, and associated clinical outcomes. The systematic literature review reveals that in mainland China, the maternal GBS colonization rate varies from 3.7% to 14.52%, and the incidence of invasive GBS disease in infants is 0.55–1.79 per 1000 live births, indicating a significant health concern with Serotype III being the most prevalent. The available data in China suggest that specific GBS serotypes are predominant in causing disease. This comprehensive analysis highlights the varied prevalence of GBS colonization among pregnant women in China and the consequent risks of invasive GBS diseases in infants, with relatively high fatality rates. Furthermore, the study underscores the potential of immunization strategies targeting pregnant women, focusing on vaccines covering the major serotypes (Ia, Ib, II, III, and V) identified, to significantly mitigate the burden of GBS infections. Kao et al. (Kao et al., 2019) focused on the clinical characteristics and impacts of emerging serotype III sequence type 17 GBS invasive infections in infants in Taiwan. The study aimed to determine serotype distribution, antimicrobial resistance, clinical features, and molecular characteristics of invasive GBS isolates. The study identifies significant variations in serotype distribution, antimicrobial resistance profiles, clinical manifestations, and molecular characteristics of invasive GBS isolates from Taiwanese infants. This research highlights the diversity of GBS serotypes affecting Taiwanese infants, each with distinct antimicrobial resistance and clinical characteristics, emphasizing the need for tailored healthcare strategies. It also suggests the importance of continued surveillance and molecular epidemiological studies to better understand and combat GBS infection in this vulnerable population. Slotved et al. (Slotved and Hoffmann, 2020) analyzed the epidemiology of invasive GBS infections in Denmark from 2005 to 2018, presenting data on serotype distribution and antibiotic susceptibility in all age groups. The study reveals a significant increase in the incidence of invasive GBS infections among the elderly in Denmark from 2005 to 2018, alongside a rise in resistance to erythromycin and clindamycin. While the incidence of early-onset and late-onset GBS disease in newborns remained stable and low, there was a notable rise in GBS infections in older adults, particularly in those aged 65 and above. Additionally, the study observed an increasing trend in antibiotic resistance among GBS isolates, underscoring the need for ongoing surveillance and tailored antibiotic stewardship programs. In contrast, Choi et al. (Choi et al., 2021) discussed recent epidemiological changes in GBS among pregnant Korean women, highlighting the evolving nature of GBS epidemiology. The study indicates an increase in GBS colonization rates among pregnant Korean women to levels comparable with those in Western countries, along with notable antimicrobial resistance. The colonization rate of GBS in pregnant Korean women is 19.8%, showing an upward trend and aligning with rates in Western countries. Additionally, there is a significant presence of antimicrobial resistance, particularly to clindamycin, erythromycin, and tetracycline, underscoring the importance of periodic and comprehensive epidemiological studies to guide prevention and treatment strategies. Additionally, Zhang et al. (Zhang et al., 2021) conducted a retrospective study in Shanxi, China, focusing on the molecular characterization of pathogenic GBS strains, with a high incidence of sequence type 10 strains in infants and pregnant women. A high prevalence of ST10 was found in both pregnant women (44.4%) and infants (72.2%) with GBS, highlighting its significant role in regional infections. The majority of GBS isolates harbored the pilus island combinations PI-1+PI-2a, indicating its potential importance in the pathogenesis and transmission of GBS, thus suggesting targets for future interventions and vaccine development.

The treatment of infections caused by MDR pathogens poses a significant challenge in clinical practice. Various studies have explored different therapeutic interventions and their outcomes in combating MDR infections. Nørgaard et al. (Nørgaard et al., 2019) conducted a systematic review to identify current antimicrobial treatment options for infections with MDR Gram-negative bacteria. The study found that monotherapy and colistin combination therapy showed clinical and microbiological success rates ranging from 70% to 100%, depending on the infection site and severity. In the context of specific bacterial infections, Liu et al. (Liu et al., 2020) investigated the influence of Autoinducer-2 (AI-2) on tetracycline resistance in *Streptococcus suis*. The study demonstrated that the addition of exogenous AI-2 led to an increase in MIC compared to the wild type strain, highlighting the importance of exploring new approaches to combating antimicrobial resistance. Furthermore, Xiangru et al. (Xiangru et al., 2023) evaluated the clinical efficacy of Buzhong Yiqi decoction (BZYQ) in the treatment of hospital-acquired pneumonia (HAP) with multi-drug-resistant bacteria (MDRB). The study reported a higher clinical success rate and pathogen eradication rate in the intervention group compared to the control group, indicating the potential of BZYQ as a treatment option for MDRB infections.

Therefore, epidemiological surveillance of GBS, assessment of PRGBS, and evaluation of multidrug-resistant genotypes are of crucial importance (Nagano et al., 2012). Antibiotic resistance in GBS with their specific targets and mechanisms are shown in Table 2.

**TABLE 2:** Summary of Antibiotic resistance in Group B *Streptococcus* with their specific targets, mechanisms and references.

Antibiotic class	Antibiotic	Mechanism of resistance	Gene(s) involved	References
Beta-Lactams	Penicillin	Mutations in penicillin-binding proteins (PBPs) leading to reduced affinity	Mutations in PBP1A, PBP2B, PBP2X (notably V405A, Q557E in PBP2X)	Chu et al. (2007), Dahesh et al. (2008), Kimura et al. (2008), Verani et al. (2010), Longtin et al. (2011), Nagano et al. (2012), van der Linden et al. (2019), Ali et al. (2022b), Beres et al. (2022), Demczuk et al. (2022), Koide et al. (2022), Uruén et al. (2022), Khan et al. (2023), Verma et al. (2023)
Macrolide, Lincosamides, Streptogramins	Erythromycin, Clindamycin, Streptogramin B	Efflux pump expulsion; Ribosomal modification by methylation; Inactivation of lincosamide antibiotics	*mef* genes; *erm* genes; *linB* and *lnu* genes	Bozdogan et al. (1999), de Azavedo et al. (2001), Lopardo et al. (2005), Shen et al. (2005), Bacciaglia et al. (2007), Savoia et al. (2008), Faccone et al. (2010), Sadowy et al. (2010), Seo et al. (2010), Back et al. (2012), Arana et al. (2014), Lu et al. (2014), Akdoğan Kittana et al. (2019), Heyar et al. (2020), Padekar et al. (2020), Santana et al. (2020), Barros, 2021; Du et al. (2021), Kumar Chaudhary and Piya (2021), Mudzana et al. (2021), Van Du et al. (2021), Bae et al. (2022), Hsu et al. (2023), Wang et al. (2023)
Ketolides	Telithromycin	Strong affinity to bacterial ribosomes; overcoming common resistance mechanisms	*ermB* gene, ribosomal protein variations	Farrell and Felmingham (2004), DiPersio and DiPersio (2006), Takaya et al. (2010), Wang et al. (2015)
Fluoroquinolones	Levofloxacin, Ciprofloxacin, Gatifloxacin, Moxifloxacin, Gemifloxacin	Mutations in QRDRs of DNA gyrase; topoisomerase IV genes (GyrA, ParC, ParE)	Triple mutations in GyrA-ParC-ParE; Double mutations in GyrA (S81L) and ParC (S79F/Y); Additional mutations in ParC (D83Y/N) and GyrB mutations	(Back et al., 2012; Wehbeh et al., 2005; Murayama et al., 2009; Wang et al., 2013; Piccinelli et al., 2015a; Piccinelli et al., 2015b; Ali et al., 2020; Hayes et al., 2020)
Tetracyclines	Tetracycline	Ribosomal protection proteins	*tetM* (predominant); *tetO*, *tetK* and *tetL*	(Piccinelli et al., 2015b; Granlund et al., 2010; Huiling et al., 2010; Jia-de, 2010; Dutra et al., 2014; Shabayek and Abdalla, 2014; Liu et al., 2021)
Glycopeptides	Vancomycin	Acquisition of vanG-like clusters	Sequences similar to vanW, vanXY, and full-length vanG (in the case of a 2658bp tandem repeat identical to vanG of *E. faecalis*)	Park et al. (2014)
Multidrug-resistant GBS	Penicillin-resistant GBS (PRGBS)	Surviving and spreading in hospital settings, leading to nosocomial infections	Not specified	(Khan et al., 2023; Talebi Bezmin Abadi et al., 2019)

## 4 GBS related clinical diseases in obstetrics and gynecology

### 4.1 GBS infection in non-pregnant women

The incidence of GBS disease is increasing in non-pregnant adults or adults with impaired immune function, especially among those with underlying conditions. Approximately 20%–70% of infections are nosocomial (Miselli et al., 2022). Several clinical diseases have been confirmed to be caused by GBS infection. The most common diseases are skin and soft tissue infections (Akbari et al., 2023), and GBS can also cause vaginal infections in non-pregnant women. This infection can cause symptoms such as vaginal inflammation (Tano et al., 2021), abnormal vaginal discharge (Dilrukshi et al., 2021), and itching (Tano et al., 2021). GBS is also one of the common pathogens that cause urinary tract infections in non-pregnant women (Balasubramanian et al., 2023). Urinary tract infections can cause symptoms such as frequent urination, urgency, and pain during urination. Although GBS infection is not usually considered a sexually transmitted disease, it can be transmitted to non-pregnant women through sexual contact (El Beitune et al., 2006). In this case, the infection may affect areas such as the vagina, cervix, and urethra. Non-pregnant individuals with compromised immune function (Bebien et al., 2012), such as those receiving immunosuppressive therapy, with chronic diseases, or undergoing chemotherapy, may be more susceptible to GBS infection. For GBS infection in non-pregnant women, the general approach includes diagnosis and laboratory testing by a healthcare professional to determine the presence of GBS infection. If the infection is confirmed, appropriate antibiotic treatment such as penicillin or other antibiotics may be prescribed to eliminate bacterial infection. Symptomatic measures such as pain relief and anti-itch treatment can also be taken.

### 4.2 GBS infection in pregnant women

GBS infection in pregnant women can manifest as asymptomatic clinical infection or progress to sepsis. GBS infection can cause maternal bacterial urinary tract infections, pyelonephritis, postpartum mastitis, and endometritis (Sundin et al., 2021). Among systemic GBS infections in mothers, serotypes Ia, III, and VI account for the majority (Barro et al., 2023). GBS infection is also associated with premature birth, premature rupture of membranes, chorioamnionitis, fetal infection, and stillbirth. Approximately 1%–3% of infected newborns will develop early-onset disease within 7 days after birth (Preventing neonatal group B streptococcal infection, 2011). The main causes of early-onset neonatal infection are vertical transmission from the mother and GBS infection of the amniotic membranes. Over 95% of early-onset infections are related to GBS serotypes Ia, Ib, II, III, IV, and V. Among newborns with early-onset infection, 80%–85% will develop sepsis (Simonsen et al., 2014), 10% will develop pneumonia (Finsterer, 2022), and 5%–10% will develop meningitis (Manzanares et al., 2023). Meningitis is a late-onset disease that occurs between 6 days and more than 90 days after birth. Currently, there is limited understanding of the pathogenesis of late-onset GBS infection (Delara et al., 2023), which may be related to vertical transmission, nosocomial infection, or community-acquired infection. Serotype III GBS is highly associated with meningitis (Hsu et al., 2021). Recent studies have shown that preterm birth is a major risk factor for late-onset GBS infection (Gonçalves et al., 2022; Choi et al., 2023; Paul et al., 2023). In addition to meningitis, clinical manifestations of late-onset infection also include bacteremia and osteoarticular infections (Raabe et al., 2019). Currently, there are no effective preventive measures for GBS infection in pregnant women. Extremely late-onset GBS infection refers to GBS infection in infants older than 3 months. The risk factors for extremely late-onset GBS infection are similar to those for late-onset infection. However, most cases of extremely late-onset GBS infection occur in preterm or extremely low birth weight infants (Liu and Tong, 2019; Suffolk et al., 2019). Infants with extremely late-onset GBS infection are more susceptible to immunodeficiency disorders. The most common clinical manifestations of extremely late-onset GBS infection are bacteremia and meningitis.

In infants infected with GBS, the mortality rate of early-onset infection is about 2%–3%, while the mortality rate of late-onset infection is about 1%–3% (Stephens et al., 2023). In premature infants, the mortality rate of early-onset GBS infection is approximately 20%–30%, and the mortality rate of late-onset infection is about 5%–8%. Although infants infected with GBS can survive, their 10-year survival rate is very low (Kalliola et al., 1999), and they often require multiple hospitalizations within the first 5 years of life. Research has found that children with GBS infection are three times more likely to die or be hospitalized within 11 years after birth (Platt and Gilson, 1994). GBS infection can increase the risk of permanent neurological disabilities such as cerebral palsy and epilepsy. 51% of infants with GBS meningitis can grow up, while 25% of infants with GBS meningitis have mild to moderate neurological disabilities, and the remaining infants with GBS meningitis will develop severe neurological or functional impairments. Therefore, early detection and prevention of GBS infection in newborns and infants is crucial.

According to the recommendation of CDC, GBS screening should be performed in pregnant women between 36 weeks 0/7 days and 37 weeks 6/7 days (approximately 5 weeks before delivery) (Verani et al., 2010). If the screening result is positive for GBS, antibiotics should be given during delivery to prevent infection. If GBS is found in the vaginal flora of pregnant women at any time, regardless of the concentration, it indicates an overgrowth of GBS. If the concentration of bacteria in the urine is higher than 105 CFU/mL at any time during pregnancy, antibiotic treatment should be given to the pregnant woman before delivery, and intrauterine injection should be performed during delivery. If the concentration of bacteria in the urine is lower than 105 CFU/mL, antibiotic treatment before delivery is not necessary, but antibiotic prophylaxis during delivery is still necessary. For pregnant women who have already given birth or have premature rupture of membranes before 36 weeks of pregnancy, antibiotic prophylaxis will be continued until the baby is born. GBS screening in pregnant women significantly reduces the incidence of early-onset GBS infection in newborns, reducing it by nearly 85% compared to no screening (Hanson et al., 2022). However, late-onset infection is not prevented.

Penicillin G is the preferred drug for prophylaxis against GBS infection due to its low cost, low toxicity, and narrow spectrum of antibacterial activity (Ikebe et al., 2023). According to the guidelines of the American Academy of Pediatrics, antibiotics should be administered at least 4 h before delivery to ensure that the concentration of Penicillin G in the amniotic fluid and placental circulation reaches a sufficient level, thereby reducing the transmission of GBS from mother to baby. If a pregnant woman is allergic to beta-lactam antibiotics, cefazolin should be used instead. Pregnant women who are sensitive to clindamycin should receive clindamycin treatment, while those who are resistant to clindamycin should receive vancomycin treatment (Duffy et al., 2022). Unlike conventional antibiotic treatment, antibiotic prophylaxis is used only as “local antibiotic treatment”. “Comprehensive antibiotic treatment” is a method used to eradicate *Helicobacter pylori* (Luo et al., 2023), which has been classified as a class I human carcinogen by the World Health Organization’s International Agency for Research on Cancer. This high-dose antibiotic therapy can eradicate *Helicobacter pylori* colonization and treat gastric cancer. However, in the field of obstetrics, this “comprehensive antibiotic treatment” cannot be implemented because it may cause serious harm to the health of both the mother and the fetus, including fatal diseases or chronic disabilities.

However, there are also certain limitations to prophylactic antibiotic treatment. Due to the risk of allergic reactions, the necessity of conducting antimicrobial sensitivity tests on pregnant women is increasingly being emphasized. Some research reports indicate that the rate at which maternal antibodies are transferred to newborns is approximately 0.5–0.7, indicating a relatively poor effectiveness of prophylactic antibiotic treatment (Dad et al., 2021).

### 4.3 GBS infection and the microbiota of pregnant women

GBS infection is closely related to the microecology of pregnant women. Microecology refers to the balance between beneficial bacteria (such as *Lactobacillus*) and other microorganisms in the human body (Mejia et al., 2023). Under normal circumstances, the vagina and intestines of healthy pregnant women may carry a certain amount of GBS, but it maintains a balance with other beneficial bacteria. However, certain factors may lead to an imbalance in microecology, causing GBS to overgrow and cause infection.

GBS primarily colonizes in the vagina, where there is a normal presence of *Lactobacillus* and other beneficial bacteria that maintain an acidic environment by producing substances such as lactic acid (Kling et al., 2009), inhibiting the growth of pathogens. When the number or balance of *Lactobacillus* in the vagina decreases or becomes imbalanced, the proliferation and risk of GBS infection increase. In addition, the gut microbiota is closely related to the microecology of other parts of the body. Imbalances in the gut microbiota can affect overall immune system function and the colonization and infection process of GBS.

During pregnancy, the immune system undergoes a series of regulatory changes to tolerate the fetus (Sweeney et al., 2020). These changes may affect the immune response to GBS infection. When immune system regulation becomes imbalanced, the risk of GBS infection may increase. Imbalances in vaginal microecology usually involve a lack of *Lactobacillus* and excessive growth of other pathogenic microorganisms (Mei and Li, 2022). Bacterial vaginosis is a mixed infection caused by an imbalance in normal vaginal flora, where *lactobacillus* is reduced and other bacteria multiply, mostly anaerobic bacteria (Kamga et al., 2019). Bacterial vaginosis and aerobic vaginitis are considered to be associated with various severe obstetric complications, such as preterm birth, miscarriage, premature rupture of membranes, fetal infection, and low birth weight infants (Choi et al., 2022). Abnormal vaginal flora can lead to cervical shortening, resulting in preterm birth. Bacterial vaginosis often accompanies an increase in GBS infection (Xiao et al., 2023). *Lactobacillus* count significantly decreases and *streptococcus* count increases in pregnant women with bacterial vaginosis (Mohammed et al., 2020). Therefore, the balance of microecology in pregnant women is crucial for preventing GBS infection.

There are some strategies that can help maintain or improve the balance of microecology in pregnant women, such as consuming foods rich in *lactobacillus* and probiotics, such as yogurt and fermented foods, which help maintain gut health (Sroka-Oleksiak et al., 2020). Excessive or inappropriate use of antibiotics can disrupt beneficial bacteria and lead to an imbalance in microecology (Pulingam et al., 2022). Antibiotics should be used under the guidance of a doctor. Long-term exposure to high-stress environments can also affect the balance of microecology. Pregnant women can reduce stress through appropriate rest, relaxation techniques, and stress management.

## 5 Prevention, detection, and treatment of GBS

### 5.1 Intrapartum antibiotic prophylaxis

Intrapartum antibiotic chemoprophylaxis (IAP) is an effective strategy for preventing early-onset neonatal GBS disease by inhibiting or reducing the colonization of GBS within the maternal genitourinary and gastrointestinal tracts, which in turn reduces the vertical transmission of GBS to the newborn (Le Doare et al., 2017). In the 1990s, the incidence of GBS-EOD (early-onset disease) in live births in the United States was 1.80 per 1,000. However, following the widespread implementation of the IAP policy, the incidence dropped significantly to 0.23 per 1,000 in 2015, representing an 80% decrease (Nanduri et al., 2019). IAP has been proven to be an effective means for preventing early-onset GBS disease in newborns. The approach to IAP currently represents the most widely used strategy to prevent GBS infections in pregnant women in many developed countries (Nuccitelli et al., 2015). The criteria for administering IAP are primarily based on the colonization status of GBS in the pregnant woman and/or an assessment of perinatal clinical risk factors. The basis for these assessments may vary among different countries or regions (Tsega et al., 2015; Wang et al., 2021).

In 1996, the American College of Obstetricians and Gynecologists (ACOG) recommended in its guidelines on preventing neonatal GBS-EOD that IAP should be determined by a combination of microbiological screening and assessment of risk factors (ACOG committee opinion, 1996). However, the revised guidelines from the U.S. Centers for Disease Control and Prevention (CDC) in 2002 emphasized the greater efficacy of microbiological screening, and recommended IAP for women with positive GBS bacteriuria, those with a history of neonatal GBS infection, or those with unknown GBS status but presenting labor risk factors (Schrag et al., 2002). Countries such as the United Kingdom and the Netherlands do not advocate for microbiological screening of pregnant women around the time of delivery, opting instead to rely on an assessment of clinical risk factors to determine whether to administer IAP (ACOG committee opinion, 1996). The decision to use risk factor assessments over microbiological screening is informed by the cost of screening tests and a desire to prevent the overuse of antibiotics (Le Doare et al., 2017).

A systematic review in 2017, which included IAP policies from 60 countries, identified the following major risk factors for prioritizing IAP: 1) Preterm birth (<37 weeks); 2) Premature rupture of membranes; 3) Prolonged duration of membrane rupture; 4) Positive GBS bacteriuria; 5) History of neonatal GBS infection; 6) Maternal fever (temperature >38°C); 7) Intra-amniotic infection. Of the 60 countries, 25 implemented an IAP policy based on clinical risk factors, and all (60/60) countries recommended IAP for women with a history of neonatal GBS infection. Most countries (23/25) recommended IAP for cases with prolonged duration after membrane rupture, premature rupture of membranes for >18 h (PROM), or maternal GBS bacteriuria (Le Doare et al., 2017).

β-Lactam antibiotics exhibit high sensitivity against GBS and have always been the drugs of choice for the prevention or treatment of GBS infections. Nevertheless, drug sensitivity monitoring data indicate that in recent years, there has been a reduction in GBS sensitivity to β-lactam antibiotics, including penicillin (Dahesh et al., 2008; Longtin et al., 2011; Metcalf et al., 2017; Yi et al., 2019), and high levels of resistance to secondary antibiotics such as erythromycin and clindamycin (Lamagni et al., 2013). Additionally, resistance to other antibiotics, like fluoroquinolones and tetracyclines, is also on the rise (Nagano et al., 2012; Wang et al., 2015).

Intrapartum intravenous administration of penicillin is the preferred IAP treatment protocol due to its efficacy (Prevention of Group B Streptococcal Early-Onset Disease in Newborns: ACOG Committee Opinion, 2020). In case of penicillin allergy, clindamycin is used. Six countries recommend cephalosporins instead of penicillin, and four South American countries and two Asian countries, concerned about the risk of clindamycin-resistant strains in penicillin-allergic patients, suggest adding vancomycin as an alternative (Le Doare et al., 2017). In the 2020 guidelines, ACOG also recommends intravenous penicillin or ampicillin as the first-line treatment. For pregnant women with a low-risk penicillin allergy or uncertain severity of allergy, cefazolin is recommended. For those with a high-risk allergic response, clindamycin treatment can be considered after confirming the GBS strain’s sensitivity to this antibiotic (Prevention of Group B Streptococcal Early-Onset Disease in Newborns: ACOG Committee Opinion, 2020).

### 5.2 Various detection methods for GBS

In the detection of tumors caused by GBS, a combined screening and diagnostic method for GBS infection and gynecological malignancies is usually used (Achten et al., 2020). The doctor will ask the patient if there are any symptoms of infection or any previous infection records. Physical examinations may include vaginal examinations, cervical smears, and endometrial biopsies to check for any abnormal signs. Cervical smears are a commonly used screening method that involves collecting cervical cells and observing them under a microscope to look for abnormal cells or lesions (Kamal, 2022). This method can help detect cervical cancer and other early abnormalities. Human papillomavirus (HPV) is closely associated with the development of cervical cancer (Sravani et al., 2023). HPV virus screening can detect HPV infections, including high-risk types of the virus (Gavinski and DiNardo, 2023). This screening method can help detect infections early and take appropriate further actions. In some cases, specific biomarkers or blood tests can be used to assess the risk or diagnose malignancies. For example, in the screening and diagnosis of endometrial cancer, the levels of CA-125 (Pourmadadi et al., 2023) and other related markers in the blood can be measured to assess the level of risk.

### 5.3 Microbial therapy

The current administration methods of antibiotics have low cost and wide applicability, making them a better method for preventing GBS infections, especially in countries with low socioeconomic status or limited resources. However, the use of antibiotics still has limitations due to the increased risk of allergic reactions and serious risks to newborns. In this regard, microbiota therapy can serve as an alternative treatment method (Nader-Macías et al., 2021). Microbiota therapy has become a hot topic in obstetrics, gynecology, and translational research fields. Some studies have reported on the treatment of gut microbiota, such as fecal microbiota transplantation (FMT) (Cheng and Fischer, 2023), for cancer treatment. In addition, a research report has shown that the composition of gut microbiota can regulate immune response mechanisms (de Vos et al., 2022), such as anti-tumor activity, thereby producing interactions between microbiota and tumors. This microbiota regulation mechanism may be direct, but its specific downstream pathways still need to be elucidated. For general microbiota therapy, known biomarkers are used as diagnostic tools to screen and monitor patients. Microbiota-based treatment methods are used to treat various diseases and are applied in different ways (Elinav et al., 2019), including dietary interventions, probiotics, prebiotics, postbiotics, bacteriophage therapy, and fecal microbiota transplantation. Each method has its advantages and disadvantages. Probiotics are considered relatively safe (Fugaban et al., 2021). However, they do not target specific diseases and only provide a temporary therapeutic response. In addition, the effectiveness of probiotic therapy depends on specific microbial colonies and the gut microenvironment (Zhao et al., 2023). Bacteriophage therapy is a highly specific targeted treatment method (Cold et al., 2020). However, an important limitation of bacteriophage therapy is its narrow host range, where a bacteriophage can only kill certain strains of the same bacteria species and cannot kill multiple strains or different bacteria species (Azam and Tanji, 2019).

These different microbiota-based treatment methods can also be applied as new approaches to treat patients with GBS infection in the vaginal microbiota. As mentioned earlier, poor vaginal microbiota is closely associated with gynecologic malignancies and adverse obstetric outcomes, and adjusting the vaginal microbiota may potentially alter the incidence of GBS infection in pregnant women. Microbiota-based treatment methods can be similar to those used for the gut microbiota. Probiotics can be used to rebalance the vaginal flora, mainly by increasing the number of *lactobacilli*. Synbiotics, which combine probiotics and prebiotics, aim to overcome the limitations of probiotics, specifically their dependence on *lactobacilli* (Calder et al., 2022). However, symbiosis may require a specific environment. Bacteriophages bind to specific receptors on bacterial cell walls and deliver engineered therapeutic materials into host cells, resulting in promising effects. Biofilm disruptors are another treatment option (Reza et al., 2019). Polymicrobial infections produce biofilms on the vaginal epithelium and generate short-chain fatty acids, ultimately increasing the vaginal environment’s pH and leading to vaginal inflammation. A report has indicated that using antibiotics alone can reduce microbial diversity and restore populations of *lactobacilli* but cannot completely destroy biofilms (Nitzan et al., 2016). Therefore, antibiotic therapy combined with biofilm disruptor adjuvants would be a more comprehensive treatment approach.

Finally, vaginal microbiota transplantation is another microbial therapy for treating vaginal diseases (Wei and Chen, 2021). In this treatment method, volunteers are recruited and undergo medical evaluations. Their vaginal microbiota is assessed through microscopic evaluations. After screening, the best vaginal microbiota is transplanted into the recipient’s vagina. Vaginal microbiota transplantation significantly alleviates patients’ symptoms and successfully restores the composition of vaginal microbiota, including increased *lactobacillus* count. However, this relatively new method still remains controversial. Therefore, full supervision should be implemented throughout the screening process to minimize the risk of potential disease transmission, especially those that may lead to antibiotic resistance in microbes.

## 6 GBS Vaccine

While GBS remains highly sensitive to first-line β-lactam antibiotics, the widespread implementation of IAP comes with an extensive use of antibiotics, which may enhance the resistance of GBS. It can also disrupt the body’s microecology, leading to an imbalance of microbial communities. Moreover, the transfer of resistance genes can result in a greater prevalence of antibiotic-resistant pathogens across humans, animals, and the environment (McGee et al., 2021). Therefore, the development of alternative interventions to replace intrapartum antibiotic treatment has become an area of keen interest. The research and application of GBS vaccines have emerged as a promising solution. Currently, there are three main types of vaccines under investigation: capsular polysaccharide vaccines, conjugate vaccine, and protein-based vaccines.

### 6.1 Preventive vaccination with GBS vaccine

In order to reduce the global incidence and mortality rate of neonatal infections related to GBS, it is crucial to develop a vaccine against GBS (Madhi et al., 2023). It is estimated that vaccinating 70% of pregnant women with a GBS vaccine could prevent nearly 50,000 deaths related to GBS infections and 170,000 cases of preterm birth each year. However, there is currently no licensed vaccine available for preventing GBS. In 2016, the World Health Organization held consultations specifically on the development of maternal immunization vaccines and declared an urgent need for a vaccine to prevent mother-to-child transmission of GBS in order to protect the health and lives of infants worldwide (Kobayashi et al., 2016). It also proposed a strategic goal of developing a safe, effective, and affordable GBS vaccine for pregnant women to prevent neonatal deaths, stillbirths, and GBS-related diseases. Currently, two GBS vaccines have entered Phase II or III clinical trials. The first is a multivalent conjugate vaccine aimed at targeting the majority of pathogenic serotypes, while the other is a protein subunit vaccine (Duke et al., 2021). The multivalent conjugate vaccine has the potential to prevent 95% of GBS infections in pregnant women, 99% of stillbirths, and 99% of neonatal GBS infections by targeting the majority of pathogenic serotypes. The protein-based vaccine approach provides broader protection against all GBS serotypes (Dominguez and Randis, 2022). Pharmaceutical companies such as Pfizer and MinervaX have been working on developing GBS vaccines. Pfizer recently announced that the U.S. Food and Drug Administration has designated their investigational GBS vaccine (Absalon et al., 2022), Bacterial GBS 6 (PF-06760805), for prevention of the six most prominent GBS serotypes that account for 98% of GBS disease cases. MinervaX is developing a GBS candidate vaccine based on traditional multivalent conjugate technology and is preparing for Phase III clinical trials (Pawlowski et al., 2022). In low- and middle-income countries, the vaccine will greatly improve the occurrence of GBS infectious diseases and make it possible to prevent the majority of GBS-related diseases (Procter et al., 2023). Despite the advantages of GBS vaccines, their limitations include high cost, lack of coverage for all GBS strains, and the possibility of resistance. Therefore, some researchers believe it is important to detect GBS before infection progresses or develops into a severe condition.

### 6.2 Capsular polysaccharide vaccines

Capsular polysaccharide (CPS) is one of the virulence factors of GBS, which enables the bacteria to evade the host’s immune response. GBS uses its capsular polysaccharide to inhibit complement deposition and resist phagocytosis by immune cells. Additionally, CPS promotes the formation of biofilms and hampers the binding of antimicrobial peptides and Neutrophil Extracellular Traps (NETs), thereby enhancing the invasive capability of GBS. Based on the antigenic components of GBS capsular polysaccharides, GBS can be classified into ten serotypes: Ia, Ib, II, III, IV, V, VI, VII, VIII, and IX (Carreras-Abad et al., 2020).

CPS vaccines refer to vaccines developed by targeting the highly expressed CPS on the surface of GBS as the antigen and conducting research on CPS-specific antibodies. Currently, the phase I and phase II clinical trials for CPS vaccines have preliminarily confirmed their safety and efficacy. However, the immunogenicity and reactogenicity of CPS vaccines are generally low. Additionally, the IgM produced does not cross the placenta, providing only short-term protection to the fetus and no significant protection to neonates. Moreover, due to considerable structural differences between the CPS of different serotypes and the absence of cross-protective effects, the immunological protection range of monovalent vaccines is limited. Consequently, CPS vaccines have not yet been adopted for clinical use (Mettu et al., 2020).

### 6.3 Conjugate vaccine

Conjugate CPS vaccines aim to enhance immunogenicity through the covalent bonding of GBS’s own capsular polysaccharide with carrier proteins, thereby inducing the production of IgG and the memory of T-cells and B-cells (Aceil et al., 2022). The early development of CPS conjugate vaccines involved the covalent attachment of tetanus toxoid (TT) to type III CPS to form a monovalent conjugate vaccine (III-TT). Currently, monovalent, bivalent, and trivalent vaccines targeting GBS serotypes Ia, Ib, II, III, and V have been researched in non-pregnant and pregnant women, demonstrating safety and efficacy in phase I and phase II clinical trials (Baker et al., 2003; Madhi et al., 2016). In 2021, Absalon et al. evaluated the safety and immunogenicity of a novel hexavalent vaccine (GBS6) for serotypes Ia, Ib, II, III, IV, and V, which proved to be safe and effective in healthy, non-pregnant adults through phase I and II clinical trials (Absalon et al., 2021). Future research will further investigate the vaccine’s effects in varying populations and its capacity to transfer antibodies to newborns.

### 6.4 Protein-based vaccine

CPS vaccines offer protection limited to specific serotypes, presenting significant constraints. On the other hand, protein vaccines are created from proteins common to all serotypes of CPS, providing a broader protective range. Moreover, protein vaccines may prevent serotype replacement or switching problems that might arise with the use of CPS vaccines (Dominguez and Randis, 2022).

Current research has been focusing extensively on protein vaccines made by fusing the N-terminus of GBS surface Alpha C (αC) protein and Rib protein to produce a vaccine (GBS-NN). In 2021, Fischer et al. published the results of a phase I clinical trial for the GBS-NN vaccine, confirming its safety and immunogenicity in healthy women (Fischer et al., 2021). Building upon this in 2022, Pawlowski et al. demonstrated that the vaccine consisting of αC-N and Rib-N induced strong and persistent IgG and IgA responses against the homotypic αC-N (Pawlowski et al., 2022). It also elicited variable immune responses to heterotypic Alpha-like proteins (Alp1∼3). The study further confirmed that the IgG elicited by the GBS-NN vaccine was predominantly IgG1, which is an effective antibody subtype transferred to the fetus during the later stages of pregnancy through the placenta. Researchers are now developing additional GBS protein vaccines based on the different structural domains of the N-terminus of Alpha-like proteins.

## 7 Conclusion

The presence of GBS implies that infants and newborns may experience severe clinical outcomes. However, for elderly individuals with GBS infection, the lethality of the infection itself is relatively low. Considering the potential role of GBS in the development of gynecologic malignancies, although GBS may not be the sole major cause, it is a key factor leading to adverse outcomes. GBS may play a crucial role in the development of severe clinical symptoms, but its detection becomes challenging due to interference from many other factors. GBS can even act as a powerful dormant pathogen, manipulating and regulating other bacteria, thereby resulting in serious clinical consequences. Therefore, it is crucial to study the interaction and impact mechanisms between GBS and bacteria and the host environment. More research is needed in the future to examine the pathogenesis and mechanisms of action of GBS, such as high-throughput sequencing technologies like RNA-seq, metagenomics, and metabolomics. In addition, professional discussions and collaborative research should be encouraged to develop better management strategies for GBS, aiming to control and reduce the maternal and infant mortality and morbidity caused by GBS infections. This comprehensive approach not only allows for better understanding of GBS but also contributes to the health of pregnant women and newborns nationwide and even globally.
