# Correction: Current research update on group B streptococcal infection related to obstetrics and gynecology

**DOI:** 10.3389/fphar.2026.1925379

**Published:** 2026-07-14

**Authors:** Ying Liu, Hao Ai

**Affiliations:** Liaoning Provincial Key Laboratory of Follicular Development and Reproductive Health, The Third Affiliated Hospital of Jinzhou Medical University, Jinzhou, Liaoning, China

**Keywords:** group B streptococcal, obstetrics and gynecology, antibiotic prophylaxis, group B streptococcal vaccine, microbial therapy

Affiliation Liaoning Provincial Key Laboratory of Follicular Development and Reproductive Health, The Third Affiliated Hospital of Jinzhou Medical University, Jinzhou, Liaoning, China was erroneously given as Liaoning Provincial Key Laboratory of Follicular Development and Reproductive Health, Jinzhou Medical University, Jinzhou, Liaoning, China.

The original version of this article has been updated.

